# Polysiloxanes in Theranostics and Drug Delivery: A Review

**DOI:** 10.3390/polym10070755

**Published:** 2018-07-09

**Authors:** Ignazio Blanco

**Affiliations:** 1 Department of Civil Engineering and Architecture, University of Catania, Viale Andrea Doria 6, 95125 Catania, Italy; iblanco@unict.it; 2 UdR-Catania Consorzio INSTM, Viale Andrea Doria 6, 95125 Catania, Italy

**Keywords:** polysiloxanes, theranostics, drug delivery, nanomedicine, PDMS, silicon

## Abstract

One of the historical problems of medicine is that often, diagnosis and therapy do not interface, at best. Moreover, especially in some areas, such as oncology, the stress for the organism during the two phases (diagnosis and therapy) can become excessive, and be fatal to the success of the treatment. The extraordinary progress of nanotechnology in the last 25 years has offered the opportunity to build a nanoplatform able to ferry drugs, and loads onto them both imaging and therapeutic functions, thus creating nanosystems capable of diagnosis, drug delivery, and monitoring of therapeutic response. The purpose of this unusual, and up to recent times, unimaginable, marriage between diagnosis and therapeutics is the reaching of protocols more specific to individuals. The dual use of particles/device lead to a personalized medicine. Due to their biocompatibility, versatility, physical and chemical resistance, and ability to be functionalized, silica nanoparticles and polysiloxanes are the heart and the shield of this nanoplatform, respectively. In this short review, I analyze the applications of these silicon-based materials in the field of controlled drug delivery.

## 1. Introduction

When we talk about silicon-based compounds, our mind flies towards the Californian valley, that has made the term silicon famous to non-chemistry people. However silicon, before becoming the most widely used element in the manufacture (sometimes at high temperature and pressure) of siloxane-based semiconductors, was the chemical base of the simplest siloxane, (SiO_2_)_n_, that is possible to find in nature in many organisms, such as structured shells, spines, fibers, and granules in many protists, diatoms, sponges, mollusks, and higher plants [1,2]. By taking inspiration from these biologically nanoscale architectures, chemistry and chemical-engineering scientists proposed, in the last 50 years, the replacing of these materials for medical and pharmaceutical applications.

Today, polymers are probably the greater and most important category of organic compounds that have changed our lifestyle, but already since the 1970s, polysiloxanes, because of their intrinsic biological nature, were used in the medical field. Huge opportunities in the design, synthesis, and modification of their physical and chemical properties have made them the most rapidly growing group of polymers, having great importance and possibilities for applications in pharmacy, medicine, and cosmetology [3].

We are writing about a very delicate field of application, perhaps the most delicate, where the keyword is compatibility, in the most severe sense of the term. From the clinical point of view, biomaterial compatibility requires many factors, among which the most important are the absence of thrombogenic, toxic, allergic or inflammatory reactions; no destruction of formed elements; no change in plasma proteins or enzymes; no immunological reactions; no carcinogenic effects; and no deterioration of adjacent tissues [4]. Coupling the biocompatibility aspects, which were written above, to their versatility (depending on the size of the molecule and on the shape of the skeleton, they can be liquids of varied viscosity, resins of varied consistence, rubbers, or elastomers), polysiloxanes are confirmed today, more than 50 years from their first applications, as one of the most used polymer categories in a wide range of biomedical applications.

The unique properties of polysiloxanes are due to the presence of both polar inorganic backbone and organic nonpolar functional groups, and as reported in many literature references [5,6,7,8], this hybrid nature is reflected in a close relationship between the structure of the polymer molecule and its physicochemical properties. The five principal structures of polysiloxanes are linear, cyclic ring polymer, branched, crosslinked, and crosslinked easily transformed into a three-dimensional network [3]. For all types of structures of polysiloxanes, four types of monomers (units) are used: monofunctional (M), difunctional (D), trifunctional (T), and tetrafunctional (Q).

Professor Pieńkowska, in her interesting review about the Pharmaceutical Applications of Polysiloxanes [3], divided these materials in four groups: non-volatile silicone fluids (linear straight chain, e.g., linear polydimethylsiloxanes, PDMS) for pharmaceutical use as an active pharmaceutical ingredient (API), and excipient, or for cosmetic use; volatile silicone fluids (typically cyclic volatile methylsiloxane, VMS), for cosmetic use; silicone elastomers intended for medical use on skin (e.g., transdermal delivery system, patches, medical use); and silicone elastomers for scar and keloid treatment.

Polydimethylsiloxane (PDMS) is an important example of this class of polymers, and as a result of their physiological inertness, good blood compatibility, low toxicity, good thermal and oxidative stability, low modulus and antiadhesive properties, good solubility in common organic solvents, good film-forming ability, fair adhesion to various substrates, and excellent resistance to chemical and irradiation degradation, we find them in a field of applications so broad that it embraces all sectors of medicine [4]. The Si–O backbone of this class of polymers, coupled with the organic nature of the functional groups, endows it with a variety of intriguing properties. The Si–O bond length is 1.64 Å, significantly longer than that of the C–C bond (1.53 Å), and the Si–O–Si bond angle is approximately 143°, much larger than the usual tetrahedral value (110°). All these aspects translate into low barrier to rotation, low rotation energy (~0) and a torsional potential significantly lower than that about C–C bonds, thus making the PDMS chain one of the most flexible known [3,9]. Furthermore, the nonpolar organic substituents (e.g., methyl group), present shorter Si–C bond, no steric hindrance, ease of reorientation, weak intermolecular forces, and opportunity to substitute other functional groups [3].

The long list of PDMS applications includes both its use as an active substance in medical devices (blood pumps, cardiac pacemaker leads, mammary prostheses, drainage implants in glaucoma, artificial skin, maxillofacial reconstruction, replacement esophagus, contact lenses, oxygenators, medical adhesives, finger joints, coatings for cochlear implants, catheters, drug delivery systems, and denture liners) [4], as well as excipient (skin adhesive patches, pressure-sensitive silicone adhesives, again controlled drug delivery systems, skin care emollients, ointments and lotions, and various auxiliary materials like contact lenses, various implants) [3]. This review aims to focus on one of the most widespread applications of this important class of polymers, drug delivery, by highlighting, where necessary, also, the possible improvements related to the interaction with the human body, because despite its bio-inertness, some problems can be recorded in this regard.

## 2. Polysiloxanes in Theranostics and Drug Delivery

### 2.1. Brief Summary of the History of Polymer Applications in the Theranostic Field

In the perspective of minimizing toxicity to the human body and increasing the drug efficiency, theranostics tries to perform diagnosis and therapy at the same time, identifying the individual tumor particles [10,11,12]. The key role is entrusted to the nanoparticles accompanied in the place of interest, inside the body, by an external magnetic field.

Regarding the diagnosis, nanoparticles can be used as agents in magnetic resonance imaging (MRI), to increase the contrast of the image and allow better detection of tumors. As for the therapy, they are used for magnetic hyperthermia, a technique based on heating by applying a weak alternating magnetic field with an appropriate frequency. In this way, the cancer cells, being more sensitive to heat than healthy ones, are destroyed, once a local temperature between 41 and 46 ° C is reached [13].

In this regard, different nanoparticles have been developed in the field of theranostics [14,15] aiming to exhibit long circulation in body fluids (ensuring greater accumulation in tumor tissues due to their active or passive targeting properties); to be rapidly eliminated through the renal route to ensure a sufficient difference in concentration between healthy and diseased zones; and to display therapeutic potential and contrast properties.

In both cases, multifunctional nanoparticles must be coated with a polymer, which possesses a series of particular characteristics, in order to protect the cores and to provide functionality (e.g., biological recognition functions, thermoresponsivity, and catalytic properties) [16]. Surface modification of nanoparticles with coupling agents can be employed to offer them better compatibility with dispersing media, to prevent nanoparticles from aggregation, as well as to render them with chemical reactivity [17,18,19]. This also avoids homogeneity and compatibility problems between the two phases, and thus, designing materials with well-adjusted properties makes their specific use possible.

3-Glycidoxypropyltrimethoxysilane (GLYMO), 3-aminopropyltriethoxysilane (APTES), and 3-methacryloxypropyltrimethoxysilane (MEMO) have been largely used as precursors for organic–inorganic hybrid sol–gel materials [14,15]. GLYMO, APTES, and MEMO have the ability to form, simultaneously, an inorganic =SieOeSi= network through hydrolysis and condensation reactions of alkoxy groups, and covalent network with polymer through the polymerization of the corresponding epoxy, amine, and acrylate groups [20]. Silane coupling agents, that form a durable bond between organic and inorganic materials, typically show two classes of functionality.

- Hydrolyzable group, typically alkoxy, acyloxy, halogen or amine. Following hydrolysis, a reactive silanol group is formed, which can condense with other silanol groups to form siloxane linkages.
- Nonhydrolyzable organic radical that may possess a functionality that imparts desired characteristics.

**Figure 1:**
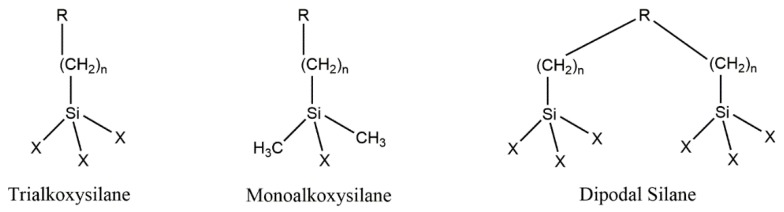
Chemical formula of silane coupling agents.

Most of the widely used organosilanes have one organic substituent and three hydrolyzable substituents (Figure 1).

Relying on the literature [17,18,19], the most promising strategies deal with the elaboration of silica-based structures that incorporate different functional entities, such as dyes for fluorescence imaging, magnetic complexes for magnetic resonance imaging (MRI), radioactive elements for scintigraphy or curietherapy, heavy elements for interacting with X- or g-rays, neutron absorbers for neutron therapy, or sensitizers for photodynamic therapy [21].

Another therapeutic application, probably the most used today, is controlled and targeted drug delivery. Also in this case, a suitable polymeric coating allows for combination of the particle with chemotherapeutic drugs that, once they reach the tumor area, can be released over time, thus allowing the administration of a lower dose and a more targeted action compared to the classic treatments. Like an airplane parachuting troops in a war zone, the polymer used to coat the actual drug plays an important role within the body.

### 2.2. Biological Applications

**Figure 2:**
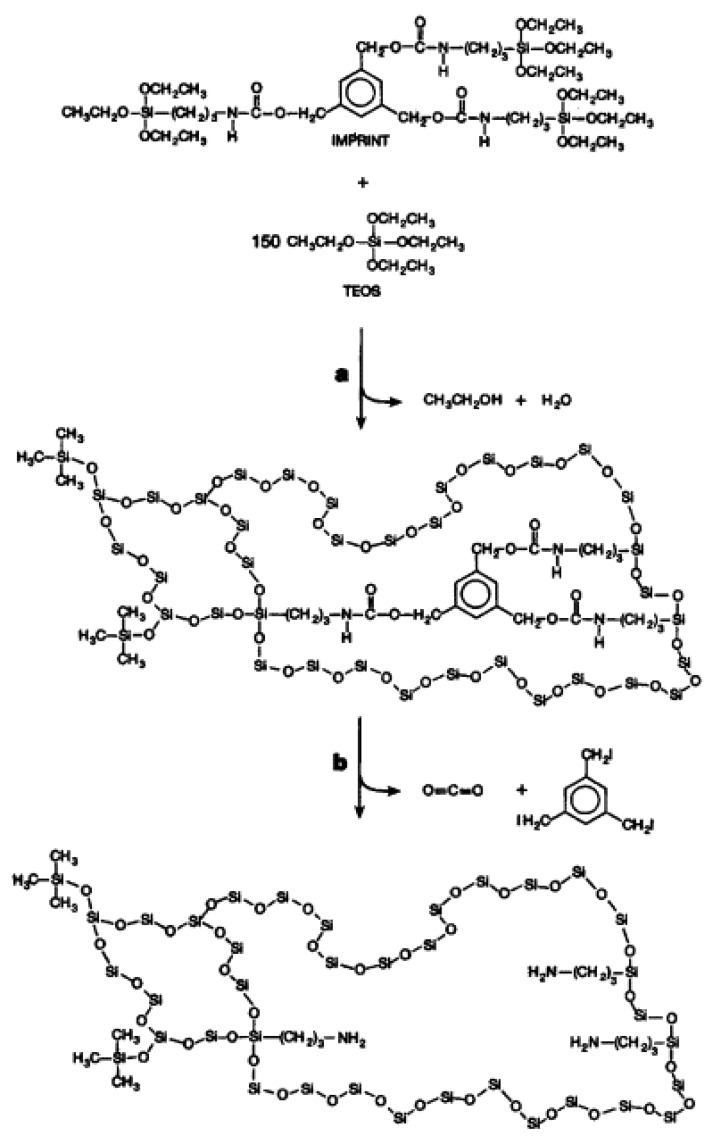
The formation of a covalent inorganic molecular imprint. (**a**) Sol–gel hydrolysis and condensation. (**b**) Removal of the covalently bound template. Reprinted from [22], © 2007 with permission from Elsevier.

Molecular imprinting is one of the biological applications of the siloxanes. This specific area has developed a method for the rapid fabrication of organic polymeric and inorganic network-structured materials that act as artificial antibodies (selectively binding a template molecule). The field has progressed rapidly, and various molecular imprints selective for protein ligands have been successfully reported. For inorganic imprints, silicon-based oxides are commonly utilized (Figure 2).

Silane derivatives are allowed to hydrolyze, typically under mildly acidic conditions, in the presence of the template to form a network sol–gel structure. Usually, a mixture of tetraethoxysilane (TEOS) and functionalized organic–inorganic ‘‘hybrid’’ alkoxysilanes, such as 3-aminopropyltriethoxysilane (APTES), is employed [22]. Hydrolysis of the TEOS generates the bulk of the rigid silica framework, and the organic functionality in the hybrid silane provides the specific interactions with the template. Intense activity elaboration of fluorescent nanoparticles for replacing the organic dyes, commonly used in biological material labelling, has been devoted by the research group of Professor Tillement. To achieve efficient protection of the Gd_2_O_3_:Tb core and covalent linkage of biotargeting groups, encapsulation by a polysiloxane shell containing amino groups appeared to be the most appropriate strategy, due to the chemical affinity between the core and the shell, which ensures a strong interaction among both components [23]. The protective coating was prepared by sequential hydrolysis of a mixture of APTES and TEOS. The latter was chosen as polysiloxane shell precursor for introduction at the outer surface of the particle amino groups, which should act as an anchoring site for biomolecules and for reticulating the oxopolymer network. They synthesized, by a two-step route, multifunctional Gd_2_O_3_ nanoparticles doped by Tb^3+^ ions (Gd_2_O_3_:Tb) and protected by a silica shell. Terbium-doped gadolinium oxide nanoparticles were obtained by applying a modified “polyol” protocol [24]. Afterward, polysiloxane shell growth was induced by hydrolysis-condensation of convenient siloxane precursors in the presence of the nanoparticles [25].

### 2.3. Common Formulations in Cancer Diagnosis and Therapy

Tan and Pfister pointed out the silicon adhesives as a critical component in transdermal drug delivery (TDD) devices, drug-loaded adhesive patches which, when applied to the skin, deliver the therapeutic agent, at a controlled rate, through the skin to the systemic circulation, and to the target organs [26]. Compared to oral or injectable dosage form counterparts, TDD presents important advantages, such as the avoidance of the first-pass metabolism by the liver and digestive system, due to the improved bioavailability of the active ingredients; a constant and monitored drug delivery; a reduced comparable dose frequency; longer therapeutic action from a single application, and reversible action (patches can be removed to reverse any adverse effects that may be caused by overdosing) [26].

**Figure 3:**
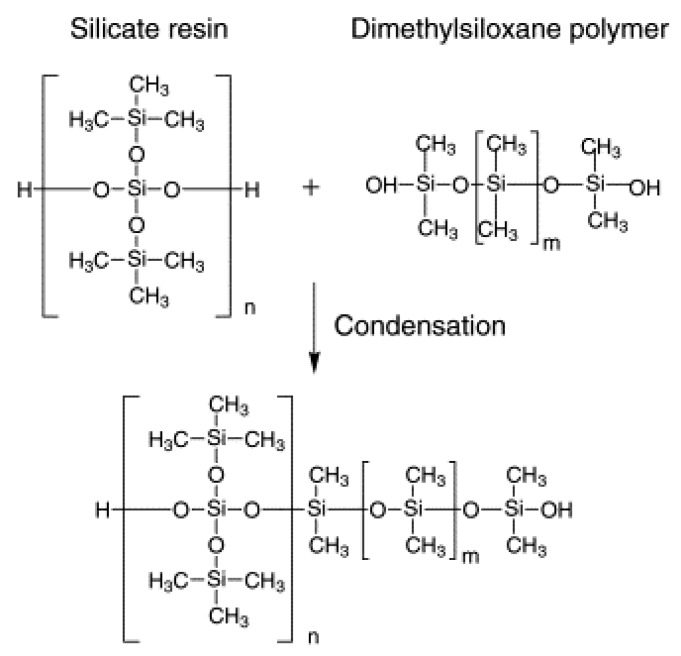
The chemistry of silicone pressure-sensitive adhesives (PSAs). Reprinted from [26], © 1999 with permission from Elsevier.

Particular requirements are essential in the choice of the material for functional adhesive, which are good biocompatibility with the skin, chemical compatibility with the drug, and effective delivery of the drug. The formulation used by Tan and Pfister was based on two major components dissolved together in a nonpolar hydrocarbon solvent: a high molecular weight PDMS containing residual silanol functionality (SiOH) on the ends of the polymer chains, and a silicate resin constituted by a three-dimensional trimethylsiloxy and hydroxyl end-blocked structure (Figure 3).

They explained their choice with the unique PDMS hybrid molecular structure and the low *T*_g_ of about −130 °C, resulting in highly flexible and extremely open macromolecular architecture with a high void volume. This structure enables silicone adhesive to have a high permeability to vapor, gases, and a wide variety of therapeutic molecules [27].

To improve cohesive strength, Bhatt and Raul proposed the incorporation of reinforcing fillers, such as finely divided silica [28], whilst to enhance the release of drugs, they managed the degree of crosslinking in the silicone polymer matrix, thus improving cohesive strength with corresponding changes in tack, adhesion, and drug release properties.

Carelli et al. used water-soluble additives, such as ethylene glycol, glycerine, and polyethylene glycols to control the water sorption into the PDMS matrix, and to enhance the release of active agents [29].

**Figure 4:**
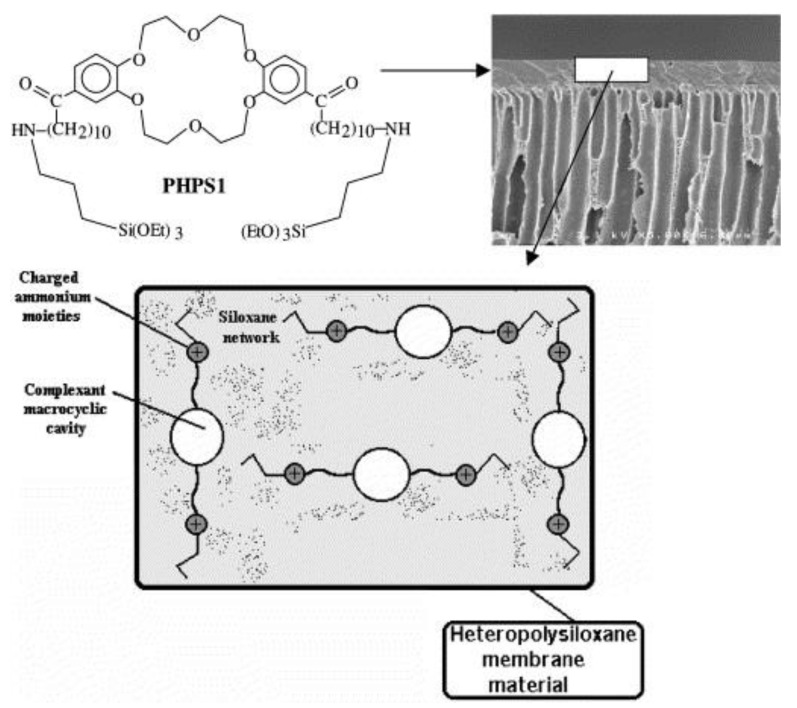
Scheme of fixed-site macrocyclic complexant membranes used in transport experiments. Reprinted from [30], © 2000 with permission from Elsevier.

By studying the dynamics and regulation of amino acid transportation in living organisms, Barboiu and collaborators tested whether fixed-site heteropolysiloxane membranes containing grafted macrocyclic receptors (Figure 4) can separate a mixture of amino acids having an intermediate configuration between liquid membranes (selective complexation by a specific carrier) and solid membranes (charge interactions) [30].

They obtained high selectivities for lipophilic acidic amino acids, although they, as well as the fluxes, decreased when the pH of the feed phase increased. The proposed membranes presented a long lifetime, which overcomes the instability of liquid membranes, resulting in a suitable alternative to the usual chromatographic methods employed for the separation of amino acids in a cascade membrane system-type.

**Figure 5:**
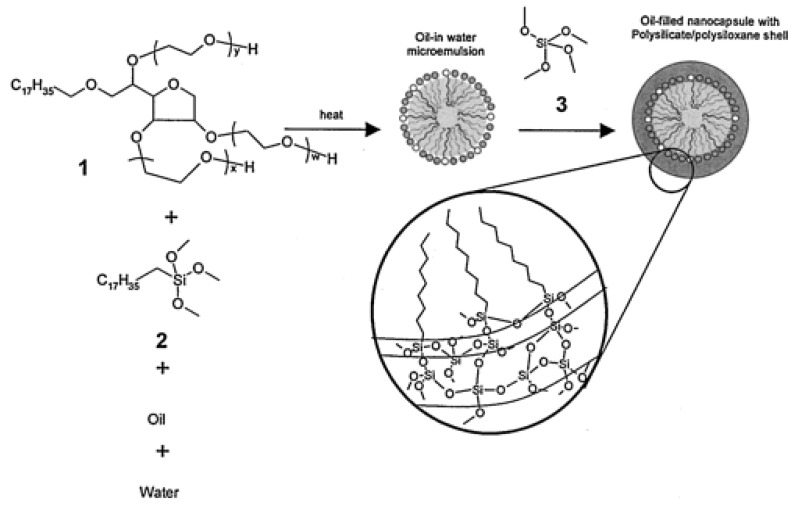
Formation of a mixed surfactant oil-in-water (O/W) microemulsion using Brij 97 ((1), filled circles, w + x + y = 10); and *n*-octadecyltrimethoxysilane ((2), open circles), followed by the formation of a stable shell through condensation of tetramethoxysiloxane (3) around the microemulsion droplet. The inset illustrates the polysiloxane/silicate network forming the shell. Reprinted from [31], © 2002 with permission from ACS Publications.

Underhill et al. synthesized oil-filled nanocapsules, with the aim to produce a system for drug detoxification therapy, using the oil droplets of an oil-in-water (O/W) microemulsion as templates obtaining a polysiloxane/silicate shell (Figure 5), imparting stability against coalescence, at the surface of the oil droplet by crosslinking *n*-octadecyltrimethoxysilane and tetramethoxysiloxane [31].

They successfully tested the nanocapsules to sequester hydrophobic compounds from saline, quinoline, and free bupivacaine. The spherical shell fortifies the microemulsion droplets against coagulation or rupture, and the dispersions have a trimodal size distribution of micelles, microemulsion droplets, and nanocapsules.

**Figure 6:**
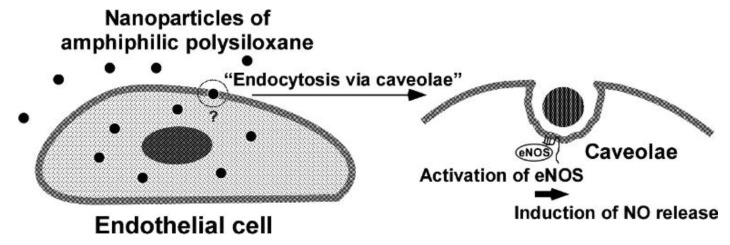
Scheme of nitric oxide release mechanisms by endocytosis via caveolae. Reprinted from [33], © 2002 with permission from ACS Publications.

Sahay et al. in their review suggested that polymers and nanomaterials can also influence intracellular transport [32], and subsequently, Nishikawa and his colleagues showed the selectively action with respect to the caveolae in human aortic endothelial cells of amphiphilic self-assembled poly(3-aminopropyl)siloxane (PAPS) of nanometric dimensions, modified with stearic acid residues and galactose [33]. They found that polysiloxane nanoparticles, endocytosed in human aortic endothelial cells, enhanced the nitric oxide release followed by the cellular uptake of the nanoparticles. The Japanese research group also confirmed that endothelial nitric oxide synthase (eNOS) was activated during cellular uptake of the nanoparticles, thus suggesting that delivery of the polymeric nanoparticles to endothelial cells can lead to the induction of nitric oxide release (Figure 6).

**Figure 7:**
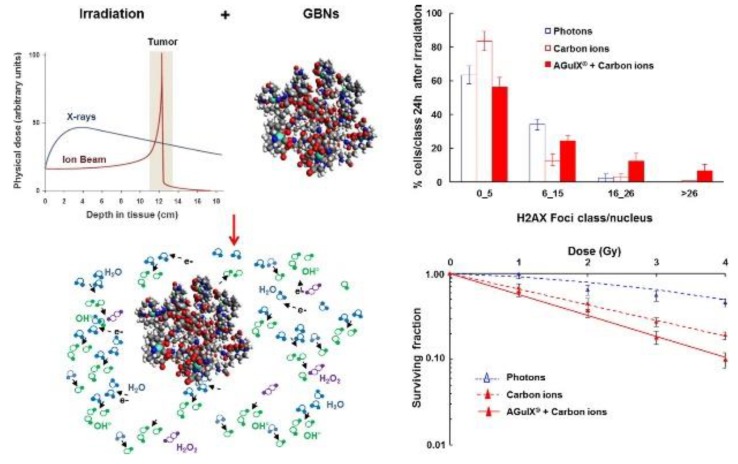
Representation of the AGuIX nanoparticle. Gadolinium-based nanoparticles AGuIX^®^ combined with photon or carbon ions exposure increase irreversible DNA double-strand breaks, leading to cell death in HNSCC cells, thus representing a promising alternative in hadrontherapy. Reprinted from [37], © 2017 with permission from Elsevier.

In the last years, an important contribution to the theranostics development was given by the group of Lyon, coordinated by the Professor Perriat and Tillement. French researchers used a polysiloxane shell for encapsulate nanometric hybrid gadolinium oxide particles (Gado-6Si-NP) for diagnostic and therapeutic applications. Quantitative biodistribution using gamma-counting of each sampled organ confirmed that these nanoparticles circulated freely in the blood pool, and were rapidly cleared by renal excretion without accumulation in liver and reticuloendothelial system (RES) uptake, enabling them to be developed as multimodal agents for in vivo imaging and theranostics in oncological applications [34]. Professor Perriat and his collaborators proposed then a top-down method consisting in the fragmentation of nanometric structures consisting of core (gadolinium oxide)–shell (polysiloxane) particles with several features and functionalities, but too large in size to escape hepatic clearance [34]. The starting structures displayed an average core size of 3.5 nm and a shell thickness of 0.5 nm. The fluorophore-encapsulated shell was rendered functionally active by modified 1,4,7,10-tetraazacyclododecane-1,4,7,10-tetraacetic acid (DOTA) ligands which are able to chelate core gadolinium ions (Figure 7). In aqueous solutions, the ligands strongly accelerate the core dissolution leading to a hollow polysiloxane sphere. This latter structure collapses and fragments into small and rigid platforms (SRPs) of polysiloxane that possess all the properties of the initial structure, bearing on their surface DOTA molecules that are partly chelated to dissolved gadolinium cations [19].

These SRP nanoparticles are excreted in a still significant proportion by the hepatic route (feces) and present an unexpectedly long circulation in the blood stream and completely evade uptake by the reticuloendothelial system. In vivo experiments with rats showed that the particles can accumulate in tumors by the electron paramagnetic resonance (EPR) effect, allowing for efficient radiotherapy guided by magnetic resonance imaging (MRI) [35,36], confirming that SRPs constitute an advance for more selective therapy and personalized medicine.

## 3. Conclusions

Theranostics has represented, for some years, the new frontier of oncology. In this short review, it has been seen how silica nanoparticles coated with siloxane polymers can be exploited both for the early detection of tumor cells and for their destruction, simultaneously performing diagnosis and therapy, as multifunctional entities. These nanoparticles allow the detection of the tumor when it has been developed only at the level of a few cells, which is impossible with simple diagnostics. It has been highlighted, both the importance of the nanoparticles’ nature and the importance of an appropriate polymer coating, which allows the drug to be anchored to the particle, which can then be released gradually over time without deteriorating its therapeutic abilities. Dual use particles/device lead to a personalized medicine, thus changing the entire healthcare scene.
